# Regional patterns of genetic diversity in swine influenza A viruses in the United States from 2010 to 2016

**DOI:** 10.1111/irv.12559

**Published:** 2019-02-13

**Authors:** Rasna R. Walia, Tavis K. Anderson, Amy L. Vincent

**Affiliations:** ^1^ Virus and Prion Research Unit National Animal Disease Center USDA‐ARS Ames IA USA

**Keywords:** genetic diversity, influenza A virus, molecular epidemiology, regional diversity, seasonality, swine

## Abstract

**Background:**

Regular spatial and temporal analyses of the genetic diversity and evolutionary patterns of influenza A virus (IAV) in swine inform control efforts and improve animal health. Initiated in 2009, the USDA passively surveils IAV in U.S. swine, with a focus on subtyping clinical respiratory submissions, sequencing the hemagglutinin (HA) and neuraminidase (NA) genes at a minimum, and sharing these data publicly.

**Objectives:**

In this study, our goal was to quantify and describe regional and national patterns in the genetic diversity and evolution of IAV in U.S. swine from 2010 to 2016.

**Methods:**

A comprehensive phylogenetic and epidemiological analysis of publicly available HA and NA genes generated by the USDA surveillance system collected from January 2010 to December 2016 was conducted.

**Results:**

The dominant subtypes and genetic clades detected during the study period were H1N1 (H1‐γ/1A.3.3.3, N1‐classical, 29%), H1N2 (H1‐δ1/1B.2.2, N2‐2002, 27%), and H3N2 (H3‐IV‐A, N2‐2002, 15%), but many other minor clades were also maintained. Year‐round circulation was observed, with a primary epidemic peak in October‐November and a secondary epidemic peak in March‐April. Partitioning these data into 5 spatial zones revealed that genetic diversity varied regionally and was not correlated with aggregated national patterns of HA/NA diversity.

**Conclusions:**

These data suggest that vaccine composition and control efforts should consider IAV diversity within swine production regions in addition to aggregated national patterns.

## 1 INTRODUCTION

Influenza A virus (IAV) causes an acute respiratory disease in swine that decreases health and welfare and results in substantial economic loss for the swine industry. Further, pigs may be susceptible to infection with avian, human, and swine IAV with the potential to generate novel reassortant viruses with zoonotic potential.^1^, ^2^, ^3^, ^4^ Consequently, control of IAV in swine is beneficial for swine and human health. Current efforts rely on whole virus inactivated vaccines or RNA vectored vaccines targeting the hemagglutinin (HA) protein to induce subtype and strain‐specific immunity. However, vaccine strategies are impaired when the strains in the vaccine do not reflect current diversity because of evolutionary processes.^5^, ^6^ Although all eight genes of the virus continuously evolve, evolution of the HA envelope glycoprotein has great potential for biologic consequence through antigenic shift (via reassortment of different gene segments), and antigenic drift (via gradual accumulation of point mutations), resulting in the cocirculation of multiple genetically diverse lineages and genetic clades.^7^, ^8^

In swine in the United States, five major H1 and H3 genetic lineages have been described. The first lineage of viruses circulating in U.S. swine emerged coincident with the 1918 human pandemic and is referred to as classical H1N1 IAV. This lineage was predominant and relatively conserved until the late 1990s when a second lineage was detected in swine. The second lineage was a novel triple‐reassortant H3N2 virus, and in addition to being maintained in U.S. swine itself, it also reassorted with endemic classical H1N1 viruses, resulting in new genetic clades of H1N1 and H1N2 viruses.^9^, ^10^ Although reassortment of HA and/or the NA segments was commonly detected, the internal gene pattern was maintained, referred to as the triple reassortant internal gene (TRIG) constellation.^11^, ^12^ The third lineage of viruses that established in U.S. swine was the result of two spillovers of human seasonal H1 viruses; these are referred to as delta‐lineage viruses.^9^, ^10^, ^13^ The fourth is the H1N1pdm09 lineage of viruses, which has been repeatedly introduced into swine herds throughout the world and has undergone reassortment with other swine IAV.^14^, ^15^, ^16^, ^17^ An additional H3 spillover from humans to swine in the early 2010s resulted in the most recent establishment of a new lineage.^18^ Given substantial expansion of genetic diversity within each of these lineages, it has become necessary to further divide the HA genes into 14 genetic clades: 5 clades of viruses within the classical‐swine lineage (H1‐α, H1‐β, H1‐γ, H1‐γ2, and H1N1pdm09); 2 clades of viruses within the δ‐lineage (H1‐δ1 and H1‐δ2); 6 clades within the 1990s H3‐lineage (IV, IV‐A, IV‐B, IV‐C, IV‐D, IV‐E, and IV‐F); and 1 clade within the 2010s H3‐lineage (human‐like H3). This nomenclature is relevant primarily in the United States, but the H1 clades described above correspond to a recently released alphanumeric global naming system for H1 HA genes (H1‐α = 1A.1 and 1A.1.1; H1‐β = 1A.2; H1‐γ = 1A.3.3.3; H1‐γ2 = 1A.3.2; H1N1pdm09 = 1A.3.3.2; H1‐δ1 = 1B.2.2, 1B.2.2.1, and 1B.2.2.2; and H1‐δ2 = 1B.2.1).^19^

Identifying patterns of genetic diversity and how they change over space and time is critical for appropriate intervention efforts. Quantifying IAV diversity in U.S. swine is challenged by the common practice of transporting swine across regions for production efficiencies. It is estimated that approximately one million pigs^20^ are moved within the United States every day and large numbers of swine also enter the United States from Canada. This movement of pigs has previously been implicated in the dissemination of the δ‐lineage from the Southeastern and South‐central United States into the Midwest.^21^ Despite this dynamic, current assessment of diversity has previously relied on national analyses of H1 and H3 viruses without separation into geographic regions; these data revealed yearly cocirculation of H1N1, H1N2, and H3N2, with H1‐δ1 (1B.2.2), H1‐γ (1A.3.3.3), and H3‐Cluster IV‐A representing the majority of HA sequences.^22^, ^23^ However, it is quite likely, given the rapid and extensive movement of swine, that the genetic diversity in one region of the United States may depend on movement patterns and that the national diversity patterns may not be evenly distributed across all states or regions. Better understanding the role of pig movement and the implications to IAV spread could facilitate surveillance efforts and provide objective criteria to help select appropriate vaccine components for improved regional control.

In 2009, following the emergence and spread of the 2009 H1N1 pandemic virus, the United States Department of Agriculture (USDA) initiated a national surveillance system to redress concerns over the quality and quantity of virologic swine IAV data.^22^, ^23^ The objectives of the surveillance are to monitor genetic evolution of IAV in swine, make isolates available for research, diagnostic reagents, and vaccine development through an IAV isolate repository, and provide publicly available sequence data for animal and human health purposes. Against this background, this manuscript describes the current genetic spatial and temporal diversity of swine IAV in the United States. We analyzed data collected through the USDA surveillance system from January 2010 to December 2016, quantified genetic diversity within and between the five USDA IAV surveillance reporting regions, determined whether aggregated national metrics of diversity are relevant at regional spatial scales, and developed four biologically informed spatial zones that may more accurately delineate U.S. IAV genetic diversity.

## 2 MATERIALS AND METHODS

### 2.1 Dataset construction and phylogenetic analysis

Nucleotide sequences for the HA, NA, and M gene segments of IAV in swine in USA collected by the surveillance system were downloaded from the Influenza Virus Resource^24^ on January 27, 2017, for the period January 2010 to December 2016. A total of 4463 HA segments, 4456 NA segments, and 4140 M segments were downloaded and analyzed, and were collected from 31 U.S. states (AL, AR, CA, CO, GA, IA, IL, IN, KS, KY, MD, MI, MN, MO, MS, MT, NC, ND, NE, NY, OH, OK, OR, PA, SC, SD, TN, TX, VA, WI, and WY). Ten virus isolates had no state information associated with them.

Sequence alignments were generated for HA‐H1 (n = 3233), HA‐H3 (n = 1230), M (n = 4140), NA‐N1 (n = 1628), and NA‐N2 (n = 2828) using default settings in MAFFT v7.222^25^, ^26^ with subsequent manual correction in MEGA7.^27^ Maximum likelihood trees for each gene alignment were inferred using IQ‐TREE v1.3.14^28^ implementing the TVM + I evolutionary substitution model that was identified via the automatic model selection function. Branch support was assessed using the ultrafast bootstrap approximation^29^ with 1000 replicates. The inferred phylogenetic trees were used to classify H1N1 and H1N2 sequences into previously defined genetic clades: H1‐α, H1‐β, H1‐γ, H1‐γ2, H1pdm09, H1‐ δ1, and H1‐ δ2.^11^, ^13^, ^22^ These clades correspond to a recently released a global nomenclature for H1 HA genes from swine IAV.^19^, ^30^ The clade designations are as follows: 1A.1 and 1A.1.1 (H1‐α), 1A.2 (H1‐β), 1A.3.3.3 (H1‐γ), 1A.3.2 (H1‐γ2), 1A.3.3.2 (H1pdm09), 1B.2.2 (H1‐δ1), 1B.2.2.1 (H1‐δ1a), 1B.2.2.2 (H1‐δ1b), and 1B.2.1 (H1‐ δ2). H3N2 sequences were assigned to Cluster IV, IV‐A, IV‐B, IV‐C, IV‐D, IV‐E, IV‐F, and human‐like H3.^18^, ^23^, ^31^ NA N1 isolates were assigned to either the classical or pandemic genetic clades,^23^ while NA N2 isolates were assigned to the 1998‐lineage or 2002‐lineage.^32^ The M gene was classified as either TRIG or pandemic.^23^, ^33^ These analyses used the resources of the USDA‐ARS computational cluster Ceres on ARS SCINet.

### 2.2 Regional analysis

The United States is divided into five different regions for IAV surveillance reporting purposes based on USDA‐APHIS veterinary services districts, with district 1 and 2 combined into one Region 1 (Figure 5C: https://www.aphis.usda.gov/aphis/ourfocus/animalhealth/animal-disease-information/swine-disease-information/ct_swine_health_monitoring_surveillance). Region 1 is comprised of Maine, Massachusetts, Rhode Island, Connecticut, New Hampshire, Vermont, New York, New Jersey, Pennsylvania, Delaware, Maryland, West Virginia, Virginia, North Carolina, South Carolina, Tennessee, Georgia, Alabama, and Florida. Region 2 is comprised of Kentucky, Ohio, Indiana, Michigan, Illinois, Wisconsin, Iowa, and Minnesota. Missouri, Oklahoma, Arkansas, Mississippi, Louisiana, and Texas make up Region 3. Region 4 comprises of North Dakota, South Dakota, Nebraska, Kansas, Montana, Wyoming, and Idaho. Alaska, Washington, Oregon, California, Nevada, Utah, Arizona, Colorado, and New Mexico make up Region 5.

Metadata for IAV in swine were collated from the Influenza Virus Resource^24^ for the period 2010—2016. Specifically, for each IAV sequence of the type H1N1, H1N2, H3N1, and H3N2, we extracted barcodes (USDA surveillance sequences are identified by a nine‐digit alphanumeric identifier in the strain name beginning with “A0”), collection date, collection site state, and serotype, and then assigned HA, NA, and M genetic clade information based upon their evolutionary history inferred from the maximum likelihood phylogenetic trees. Scripts were written in the R programming language v3.1.2^34^ to analyze the data by region and visualized using the ggplot2 package.^35^

To quantify genetic diversity within each USDA reporting region and nationally, we calculated Shannon's diversity index:

$$H^{\prime }=-\sum_{i\;=\;1}^{R}p_{i}\ln p_{i}$$

where *p*_*i*_ is the proportion of genetic clade *i* relative to the total number of genetic clades (*p*_*i*_), and after calculation this value is multiplied by the natural logarithm of this proportion. This metric quantifies the uncertainty in predicting the HA/NA type of a sample taken at random from the dataset and accounts for the abundance and frequency of unique HA/NA pairings, with high values suggesting more diversity in a region and lower values suggesting more homogeneous HA/NA pairings. This index was calculated for each year from 2010 to 2016, for each USDA reporting region and for the data aggregated to the national level. To determine the relationship between diversity of the 4 regions (Region 5 was not included because of data limitations, n = 14 from 2010 to 2016) and nationally, we conducted hierarchical clustering on the Kendall rank correlations of distances between indices calculated separately for each year. Following these analyses using bureaucratic USDA reporting regions, we conducted a similar analysis to determine if U.S. states could be grouped into zones with more similar HA/NA pairing. First, we clustered the observed data from 2010 to 2016 using data from those states that comprised ≥1% of the total data. Second, we calculated Shannon's diversity indices from the HA/NA counts for those states, and then used distances between the diversity indices to perform hierarchical clustering using ward's method for linkage.

### 2.3 Time series analysis

To study seasonal patterns of IAV in swine in the United States, we conducted time series analysis using the number of influenza isolates aggregated by month from January 2010 to December 2016. The time series was decomposed using the ts and decompose functions in the forecast package^36^, ^37^ in R v3.1.2.^34^ The additive time series decomposition was used because seasonal variation for the data was constant over time.

## 3 RESULTS

### 3.1 HA, NA, and M evolutionary trends in swine IAV

During the study period, a total of 4458 isolates were analyzed, out of which 35% were H1N1 viruses, 36% were H1N2 viruses, and 26% were H3N2 viruses. A very small percentage of virus isolates were H3N1 (0.4%) or mixed subtype (3.3%), and one HA‐H1 virus did not have an NA sequence to subtype (Figure S1). We excluded mixed subtype viruses and the single H1 virus that did not receive NA‐typing. Ten virus isolates had no state information available and were also excluded from analyses. Following removal, our detailed spatial and temporal analyses considered 4298 virus isolates. The most common genetic clades and HA/NA pairings, with global H1 nomenclature provided in parentheses, in the United States between the years 2010 and 2016 were H1‐γ (1A.3.3.3)/N1‐classical (29%), H1‐δ1 (1B.2.2)/N2‐2002 (27%), and H3‐Cluster IV‐A/N2‐2002 (15%) (Figure S2). An additional 30 types of HA/NA pairings were detected, but of these, only four HA/NA combinations—H1‐δ1 (1B.2.2)/N2‐1998, H1pdm09 (1A.3.3.2)/N1‐pdm, H3‐Cluster IV‐B/N2‐2002, and H3‐human‐like/N2‐2002—were detected at proportions greater than 2%. From 2010 to 2015, the prevalence of the pandemic M gene increased from 70% to 100%: given this trend, the USDA swine IAV surveillance terminated regular sequencing of the M gene in 2016.

To understand seasonal patterns in swine IAV, we aggregated the sequenced subtypes by month (Figure S3) across the 7 years of our study (2010‐2016). These data revealed year‐round detection of swine IAV in clinical respiratory submissions, with a primary epidemic peak in October‐November of each year and a secondary epidemic peak in March‐April of each year (Figure S3C).

### 3.2 Spatial patterns of IAV subtype and HA genetic diversity

There were 3125 H1N1 and H1N2 viruses collected from 2010 to 2016. Region 1 reported a total of 703 sequences of which 38.4% were H1N1, 34.4% were H1N2, and 27.2% were H3N2. Of the 2877 viruses submitted by Region 2, 37% were H1N1, 36.1% were H1N2, 0.35% were H3N1, and 27% were H3N2 viruses. There were 243 Region 3 isolates of which 29.2% were H1N1, 43.2% were H1N2, 2.1% were H3N1, and 26% were H3N2. There was a total of 461 isolates from Region 4, with 32% H1N1, 42.1% H1N2, 0.22% H3N1, and 26% H3N2.

**Figure 1:**
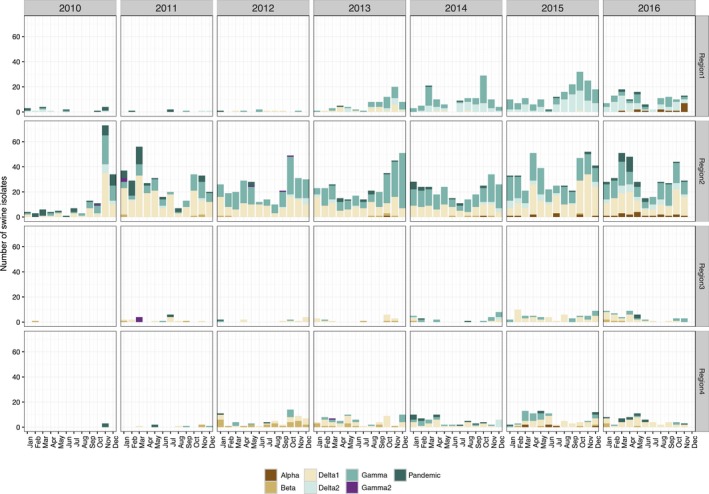
Temporal and regional patterns in H1 swine influenza A in the United States from 2010 to 2016 within the USDA Influenza A virus Swine Surveillance System. H1 genes were classified to phylogenetic clades: H1.alpha (1A.1 or 1A.1.1), H1.beta (1A.2), H1.gamma (1A.3.3.3), H1.pdm (1A.3.3.2), H1.delta1 (1B.2.2), H1.delta2 (1B.2.1) and are presented by year and by USDA‐APHIS veterinary service reporting districts (Region 5 was omitted due to insufficient data)

Of 307 H1‐δ2 (1B.2.1) viruses overall, 63% (n = 194) were from Region 1 (Figure 1). There was a steady increase in H1‐δ2 viruses detected in Region 1 since 2013 (8 viruses prior to 2012, 12 viruses in 2013, 42 viruses in 2014, 77 viruses in 2015, and 55 viruses in 2016). The remaining H1 viruses in Region 1 were classified as H1‐α (n = 15, 1A.1 and 1A.1.1), H1‐γ (n = 247, 1A.3.3.2), H1pdm09 (n = 25, 1A.3.3.2), and H1‐δ1 (n = 31, 1B.2.2). Region 2 had consistent detections of H1‐δ1 (42% 1B.2.2), H1‐δ2 (5% 1B.2.1), H1‐γ (43% 1A.3.3.3), and H1pdm09 (7% 1A.3.3.2) viruses every year from 2010 to 2016. H1‐α (1A.1 and 1A.1.1) viruses in Region 2 were detected in 2013 and gradually increased from 1 in 2013 to 19 detections in 2016. H1‐β (1A.2) viruses were consistently detected in Region 2, but represent a small fraction of all data (n = 17 from 2011 to 2015). The H1 genetic clades predominant in Region 3 were H1‐δ1 (42% 1B.2.2) and H1‐γ (20% 1A.3.3.3), with low detections of H1‐γ2 (n = 4 in 2011, 1A.3.2), H1‐β (n = 11, 1A.2), H1‐δ2 (n = 3 in 2014, 1B.2.1), and H1pdm09 (n = 8, 1A.3.3.2). In order of increasing frequency, the predominant H1 viruses in Region 4 (total of 461) were H1‐β (n = 49, 1A.2), H1‐γ (n = 74, 1A.3.3.3), and H1‐δ1 (n = 171, 1B.2.2), with low frequencies of H1‐γ2 (1 detection in 2013, 1A.3.2), H1‐α (n = 6 in 2015, 1A.1 and 1A.1.1), H1‐δ2 (n = 6 in 2014, 1B.2.1), and H1pdm09 (n = 35, 1A.3.3.2).

**Figure 2:**
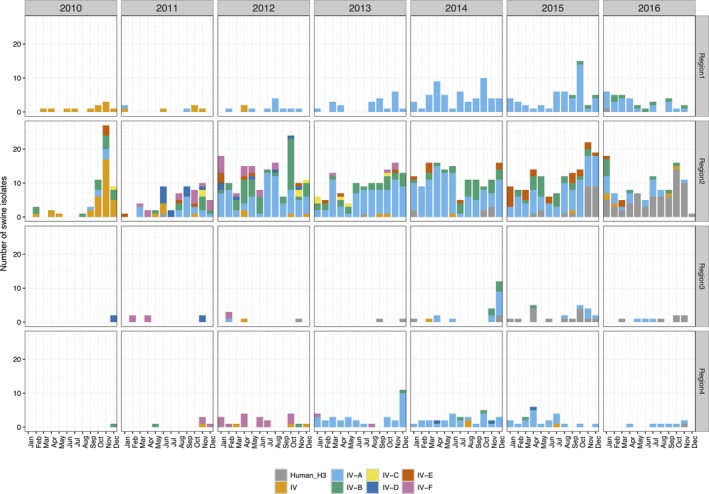
Temporal and regional patterns in H3 swine influenza A in the United States. Swine H3 isolates collected from 2010 to 2016 within the USDA Influenza A virus Swine Surveillance System were classified to H3 phylogenetic clade (Cluster IV‐A through F and human‐like H3) and are presented by year and USDA‐APHIS veterinary service reporting districts (Region 5 was omitted due to insufficient data)

The H3 subtype viruses (Figure 2) detected in Region 1 were predominantly Cluster IV (9.4%, in 2010, 2011, and 2012) and Cluster IV‐A (84% in 2013‐2016), with infrequent detections of Cluster IV‐B viruses in the later part of 2015 and 2016 (n = 12) and human‐like H3 viruses (n = 1 in 2016). Region 2 had the highest diversity of H3 genetic clades with consistent detection of Cluster IV‐A (47%), Cluster IV‐B (20%), Cluster IV (7%), and Cluster IV‐E (6%). There were detections of Cluster IV‐C (n = 11, detected from 2010 to 2013), Cluster IV‐D (n = 18, detected in 2011 and 2012), and Cluster IV‐F (n = 32, from 2011 to 2013), however, these clades were not reported in 2014, 2015, or 2016. Of note is the increase in detections of human‐like H3 viruses in Region 2 (n = 7 in 2014 to n = 23 in 2015 and n = 67 in 2016). Region 3 H3 viruses consisted of human‐like H3 (40%, increasing from n = 3 in 2014 to n = 15 in 2015) and Cluster IV‐A (33%), with infrequent detections of Cluster IV‐B (n = 6, detected in 2014 and 2015), Cluster IV‐D (n = 4, in 2010 and 2011), Cluster IV‐F (n = 6, in 2011 and 2012), and Cluster IV (n = 2, in 2012 and 2014). In Region 4, Clusters IV‐A (67%), IV‐F (18%, detected in 2011‐2013), IV‐B (6%, detected in all years except 2016), IV (6%, detected in all years except 2010, 2013, and 2016), and IV‐D (3%, detected in 2014 and 2015) were detected. There was a single detection of a human‐like H3 virus in Region 4 in 2016.

### 3.3 Spatial patterns of HA and NA pairing

**Figure 3:**
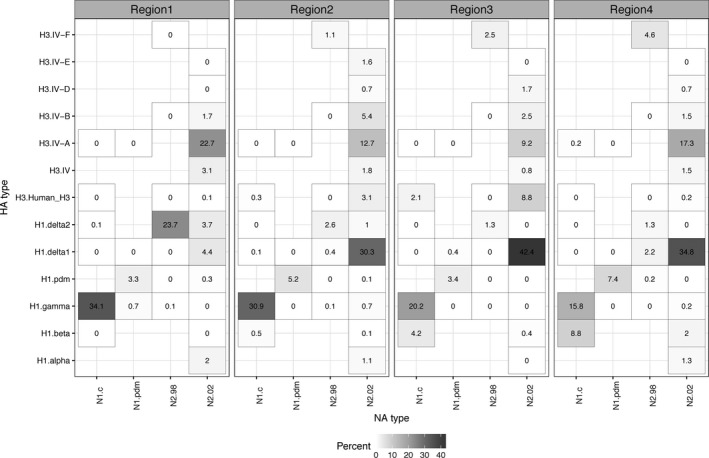
Representation of hemagglutinin (HA) and neuraminidase (NA) genetic clade pairings per virus found within the USDA Influenza A virus Swine Surveillance System from 2010 to 2016. The data are presented as percentages that were calculated based upon phylogenetic analysis and classification of HA/NA data to the following genetic clades: N1.c (N1 classical), N1.pdm (N1 pandemic), N2.98 (1998‐lineage N2), N2.02 (2002‐lineage N2), H3.Human_H3 (human‐like H3), H1.alpha (1A.1 or 1A.1.1), H1.beta (1A.2), H1.gamma (1A.3.3.3), H1.pdm (H1pdm09, 1A.3.3.2), H1.delta1 (1B.2.2), H1.delta2 (1B.2.1), H3.IV‐A, H3.IV‐B, H3.IV‐D, H3.IV‐E, and H3.IV‐F. Regional designations 1 through 5 reflect USDA‐APHIS veterinary service IAV‐S reporting districts (Region 5 was omitted due to insufficient data)

We next evaluated the patterns of HA and NA pairings in viruses by region. The most common HA/NA combination in Region 1 during 2010‐2016 was H1‐γ (1A.3.3.3) paired with N1‐classical (34.1%), followed by H1‐δ2 (1B.2.1) paired with N2‐1998 (23.7%), and H3‐Cluster IV‐A paired with N2‐2002 (22.7%) (Figure 3). About 30.9% of Region 2 viruses had an HA/NA combination of H1‐γ (1A.3.3.3)/N1‐classical, 30.3% H1‐δ1 (1B.2.2)/N2‐2002, 12.7% H3‐Cluster IV‐A/N2‐2002, followed by H3‐Cluster IV‐B/N2‐2002 and H3‐Cluster IV‐B/N2‐2002 (5.4% and 5.2%, respectively). In Region 3, 42.4% of the pairings were H1‐δ1 (1B.2.2)/N2‐2002, 20.2% were H1‐γ (1A.3.3.3)/N1‐classical, followed by H3‐Cluster IV‐A/N2‐2002 (9.2%), and H3‐human‐like/N2‐2002 (8.8%). The most common pairings in Region 4 were H1‐δ1 (1B.2.2)/N2‐2002 (34.8%), H3‐Cluster IV‐A/N2‐2002 (17.3%), and H1‐γ (1A.3.3.3)/N1‐classical (15.8%). Each region had an additional 10‐17 unique HA/NA pairings that ranged in detection frequency from 0.4% to 8.8% (Figure 3) that were not replaced by viruses with the dominant HA/NA clades.

Unique regional findings included Region 4 with 8.8% H1‐β (1A.2)/N1‐classical detections. The H1‐δ2 (1B.2.1)/N2‐1998 pairing was most frequently detected in Region 1 (23.7%), with very low detections in Regions 2, 3, and 4. Region 3 had the largest proportion of H3‐human‐like/N2‐2002 (8.8%), with low frequency detection in Regions 1 (0.1%), 2 (3.1%), and 4 (0.2%). Region 3 was the only area to not report H1‐α (1A.1 and 1A.1.1)/N2‐2002 pairing. H1pdm09 (1A.3.3.2)/N1‐pdm viruses were detected in all regions, but at slightly higher frequencies in Regions 2 (5.23%) and 4 (7.44%). Regions 2, 3, and 4 had high detections of H1‐δ1 (1B.2.2)/N2‐2002 (30.31%, 42.44%, and 34.79%, respectively) while this HA/NA pairing was detected infrequently in Region 1 (4.39%).

### 3.4 Temporal patterns of HA and NA combinations

**Figure 4:**
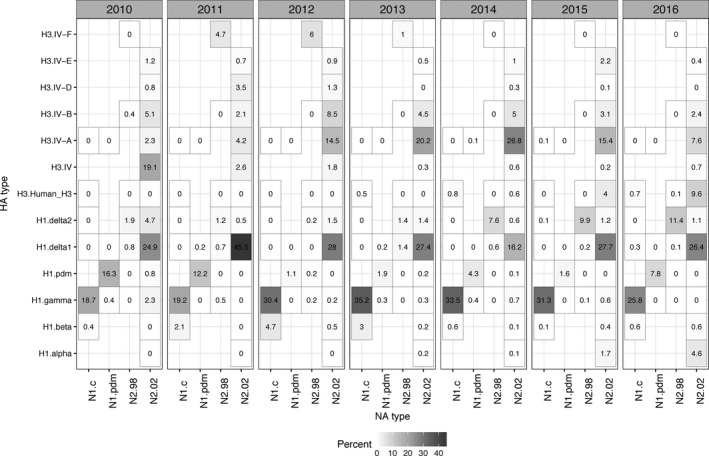
Representation of hemagglutinin (HA) and neuraminidase (NA) genetic clade pairings found within the USDA Swine Influenza A Surveillance System within each year from 2010 to 2016. The data are presented as percentages that were calculated based upon phylogenetic analysis and classification of all HA/NA data to the following genetic clades: N1.c (N1 classical), N1.pdm (N1 pandemic), N2.98 (1998‐lineage N2), N2.02 (2002‐lineage N2), H3.Human_H3 (human‐like H3), H1.alpha (1A.1 or 1A.1.1), H1.beta (1A.2), H1.gamma (1A.3.3.3), H1.pdm (H1pdm09, 1A.3.3.2), H1.delta1 (1B.2.2), H1.delta2 (1B.2.1), H3.IV‐A, H3.IV‐B, H3.IV‐D, H3.IV‐E, and H3.IV‐F

Due to the apparent changes in detection of HA and NA pairs over time, we next analyzed annual HA/NA patterns for all reporting states. Year to year fluctuations were apparent. The top four predominant viruses in 2014 were H1‐γ (1A.3.3.3)/N1‐classical (33.5%), H3‐Cluster IV‐A/N2‐2002 (26.8%), H1‐δ1 (1B.2.2)/N2‐2002 (16.2%), and H1‐δ2 (1B.2.1)/N2‐1998 (7.6%); in 2015 were H1‐γ (1A.3.3.3)/N1‐classical (31.3%), H1‐δ1 (1B.2.2)/N2‐2002 (27.7%), H3‐Cluster IV‐A/N2‐2002 (15.4%), and H1‐δ2 (1B.2.1)/N2‐1998 (9.9%), whereas in 2016, H1‐δ1 (1B.2.2)/N2‐2002 (26.4%), H1‐γ (1A.3.3.3)/N1‐classical (25.8%), H1‐δ2 (1B.2.1)/N2‐1998 (11.4%), and H3‐human‐like/N2‐2002 (9.6%) were the predominant viruses (Figure 4).

Year to year variation was apparent. The H1‐γ (1A.3.3.3)/N1‐classical pairing was one of the most abundant HA/NA combinations detected every year and remained relatively stable each year (Figure 4). In comparison, the H1‐δ1 (1B.2.2)/N2‐2002 that represented ~25% of HA/NA combinations detected over the entire study period varied from almost 50% in 2011 to only 15% in 2014. The H1‐α (1A.1 and 1A.1.1)/N2‐2002 pairing emerged in the United States in 2013 and was detected with increasing frequency every year since 2013. H1pdm09 (1A.3.3.2)/N1‐pdm was detected in higher frequency in 2010‐11 and continued in subsequent years but at much lower frequencies.^17^

The major HA/NA combination for H3 was Cluster IV‐A/N2‐2002, increasing from 2% in 2010 to 27% in 2014, and then dropping slightly to 15% in 2015 and 8% in 2016. The H3‐Cluster IV‐B/N2‐2002 and H3‐Cluster IV‐E/N2‐2002 were detected at low frequency but consistently across the years. H3‐Cluster IV‐F/N2‐1998 was not detected since 2013. The 2010‐lineage human‐like H3 virus paired with either the N1‐classical or the N2‐2002 was detected in increasing numbers from 0.5% in 2013 to ~10% in 2016.

### 3.5 National and regional comparisons

**Figure 5:**
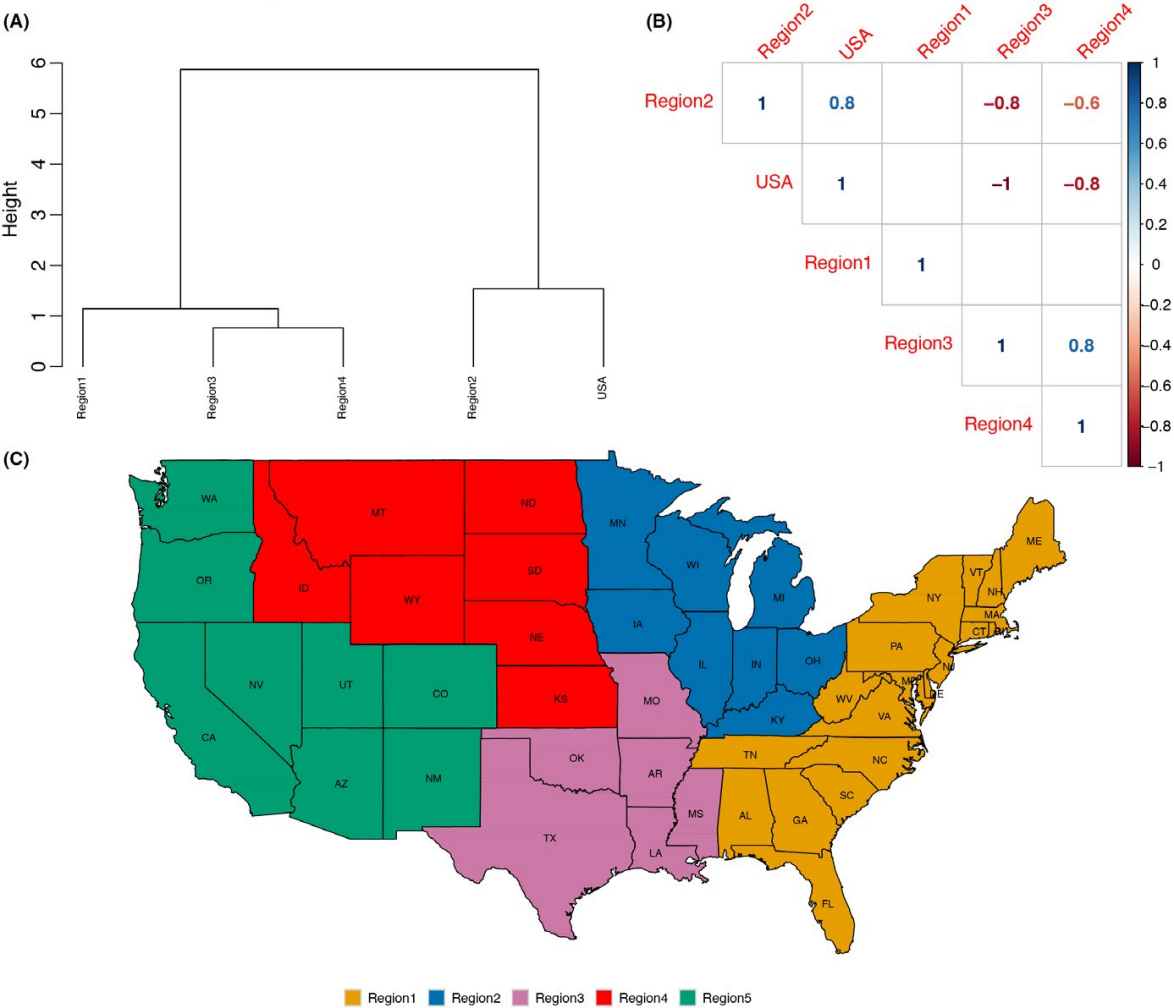
Correlation of genetic diversity between national and USDA‐APHIS Veterinary Services reporting districts. A, Hierarchical clustering dendrogram of distances between diversity indices calculated for the whole of United States and the different regions. A complete agglomeration method was implemented. B, Kendall rank correlation plot demonstrating the association between diversity aggregated to a national level (USA) vs diversity delineated to Regions 1 to 4 of USDA‐APHIS veterinary service IAV‐S reporting districts (Region 5 was omitted due to insufficient data). Correlations with *P*‐values >.01 are not considered significant and are depicted as blank cells in the correlation plot, as seen with Region 1

Because of the difference in relative proportions observed between regions (Figure 3), we next compared national and regional diversity. We calculated Shannon's diversity indices by year for the entire United States and by region (Table 1). Region 2 was the most diverse region every year, followed by Region 1 and Region 4: Region 3 was the least diverse. At the national scale, there was an almost linear increase in diversity from 2010 to 2016; the only anomaly was 2015 with higher diversity nationally and individually within each region (Table 1). Overall, the HA/NA pairings found in Region 2 mirrored what was seen nationally, but there was much less concordance between the diversity evident in Regions 1, 3, and 4 and the national descriptions of HA/NA pairings. Specifically, the HA/NA pairings in Region 2 were similar to the national pattern, whereas Region 1 clustered separately from all the other regions (Figure 5A), and Regions 3 and 4 were similar to each other but distinct from Regions 1, 2 and the national metrics (Figure 5B).

**Table 1:** Shannon's diversity indices calculated for the USA and for each USDA‐APHIS veterinary reporting region from 2010 to 2016

	2010	2011	2012	2013	2014	2015	2016
Region 1	0.10	0.05	0.07	0.25	0.38	0.46	0.37
Region 2	0.51	0.70	0.77	0.73	0.75	0.90	0.82
Region 3	0.01	0.07	0.06	0.06	0.15	0.21	0.16
Region 4	0.02	0.05	0.23	0.27	0.25	0.28	0.18
USA	0.59	0.78	0.93	0.96	1.06	1.23	1.11

**Figure 6:**
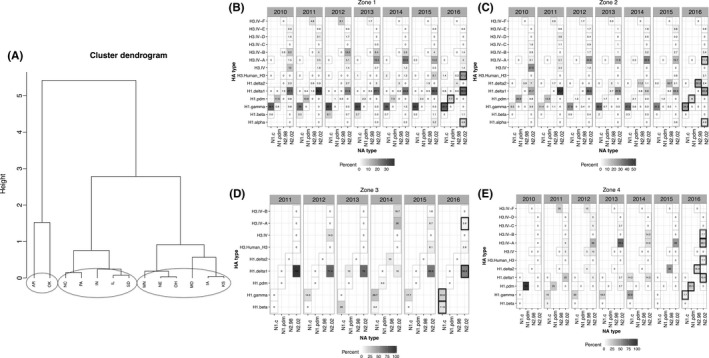
Spatial clustering of hemagglutinin (HA) and neuraminidase (NA) pairs into zones based on state‐level similarity. A, Hierarchical clustering dendrogram of distances between diversity indices calculated for those states whose data were ≥1% of the total data for the years 2010‐2016. A Ward's linkage method determined 3 clusters of states (circled) defined based upon the similarity of HA/NA pairings. Zone 1 consisted of IA, KS, MO, MN, NE, and OH; Zone 2 consisted of IL, IN, NC, PA, and SD; Zone 3 consisted of AR and OK; Zone 4 contained miscellaneous states that represented <1% of the data (not visualized) and consisted of AL, CO, GA, KY, MD, MI, MS, MT, ND, NY, OR, SC, TN, TX, VA, and WY. The HA/NA pairing data were then parsed into the Zones and visualized temporally. Temporal and spatial patterns were apparent. B, HA/NA pairings for Zone 1. C, HA/NA pairings for Zone 2. D, HA/NA pairings for Zone 3. E, HA/NA pairings for Zone 4. The bold boxes highlight the four most predominant viruses in 2016 for the given zone and the double‐lined boxes highlight the viruses that occur with lower frequency in each zone

To determine if the USDA reporting regions represented the HA/NA patterns of states within each region, we performed a cluster analysis of state‐level IAV genetic diversity data. This analysis suggested that swine IAV HA/NA diversity from 2010 to 16 was best described by 4 zones: zone 1 contained IA, KS, MO, MN, NE, and OH; zone 2 contained IL, IN, NC, PA, and SD; and zone 3 contained AR and OK (Figure 6A). Each of the states within these zones had HA/NA pairings that were more similar to each other than between clusters. We created a separate non‐statistically supported category for the remaining states whose data individually made up <1% of the total data (AL, CO, GA, KY, MD, MI, MS, MT, ND, NY, OR, SC, TN, TX, VA, and WY), a miscellaneous zone 4. The most abundant HA/NA pairings for zone 1 in 2016 were as follows: H1‐γ (1A.3.3.3)/N1‐classical, H1‐δ1 (1B.2.2)/N2‐2002, H3‐human‐like/N2‐2002, and H1‐pdm (1A.3.3.2)/N1‐pdm (Figure 6B); zone 2 were H1‐γ/N1‐classical (1A.3.3.3), H1‐δ1 (1B.2.2)/N2‐2002, H1‐δ2 (1B.2.1)/N2‐1998, and H3‐Cluster IV‐A/N2‐2002 (Figure 6C); zone 3 were H1‐δ1 (1B.2.2)/N2‐2002, H1‐γ (1A.3.3.3)/N1‐classical, H1‐β (1A.2)/N1‐classical, and H3‐Cluster IV‐A/N2‐2002 (Figure 6D); and the miscellaneous zone 4 was H1‐pdm (1A.3.3.2)/N1‐pdm, H3‐Cluster IV‐A/N2‐2002, H1‐δ1 (1B.2.2)/N2‐2002, and H1‐δ2 (1B.2.1)/N2‐1998 (Figure 6E). All zones maintained minor HA/NA pairings whose frequencies varied from year to year, but the relative proportions by year were zone‐dependent.

## 4 DISCUSSION

The purpose of this study was to characterize the genetic diversity of IAV in swine circulating in the USA based on data submitted through the USDA surveillance system. This analysis of 7 years of data revealed year‐round circulation of three subtypes (H1N1, H1N2, and H3N2), with a primary epidemic peak in October‐November, and a secondary peak in March‐April. This provides further support for biphasic seasonal outbreaks of swine IAV resulting in clinical disease similar to that reported from 2010 to 2012.^23^. In human influenza outbreaks, seasonal patterns of IAV transmission have been associated with climatic and environmental factors,^38^, ^39^, ^40^ particularly low humidity and low temperatures.^41^, ^42^ Although our data are suggestive of a similar mechanism, additional epidemiologic and experimental studies that determine the relationship between IAV in swine and climatic and environmental conditions such as temperature and humidity are necessary. A five‐year active IAV surveillance study in a single production system demonstrated a negative correlation between herd‐level IAV prevalence found in one month to the absolute humidity and outdoor air temperature found in the prior 0‐3 months.^43^ These seasonal patterns in swine support consideration of whole herd vaccination several weeks prior to each biphasic peak.

Our comprehensive phylogenetic analyses of 4463 HA segments and 4456 NA segments revealed that over the entire time period, approximately 71% of the HA sequence data could be classified into 3 major genetic clades, H1‐γ (1A.3.3.3), H1‐δ1 (1B.2.2), and H3‐Cluster IV‐A. Combining the HA data with the NA data from all contributing states, the H1‐γ/N1‐classical, H1‐δ1/N2‐2002, and H3‐Cluster IV‐A/N2‐2002 HA/NA pairings were the most abundant and were consistently detected from 2010 to present. However, when we partitioned the genetic data spatially and temporally, there were strong regional patterns in diversity and distinct changes in HA/NA pairings over time. These data demonstrated that aggregated national measures and descriptions of genetic diversity can under‐ or over‐estimate the abundance of certain genetic clades at the regional or state scales. Swine production systems are generally organized by production stage, and growing pigs are frequently transported to different locations to reduce feed costs or improve access to markets. This process results in the frequent transport of swine across state or regional boundaries, and phylodynamic techniques^44^ have demonstrated the long‐distance dispersal of swine also disseminates novel IAV clades.^21^, ^45^ However, our data suggest that the US swine herd is not endemic with a homogenous population of IAV, instead our data suggest that most IAV transmission and diversity is regional with some mixing due to pig movement, consistent with Kyriakis et al.^46^ This is supported by the consistent detection of the major HA/NA clades in all regions, along with detection of minor genetic clades and subtypes within specific regions. This represents a distinct strength of the long‐term USDA passive surveillance system data in that these data are now able to capture regional circulation and diversity, along with providing a baseline to identify the emergence of novel genetic clades.^18^, ^45^

Nationally, the predominant viruses detected from 2010 to 2016 were H1‐γ/N1‐classical, H1‐δ1/N2‐2002, and H3‐Cluster IV‐A/N2‐2002 (Figure S2). While the national pattern generally held true in Region 2, the predominant viruses in Region 1 in order of proportion were H1‐γ/N1‐classical, H1‐δ2/N2‐1998, and H3‐Cluster IV‐A/N2‐2002, and the 3 most predominant viruses in Region 3 were H1‐δ1/N2‐2002, H1‐γ/N1‐classical, and H3‐Cluster IV‐A/N2‐2002, and in Region 4 were H1‐δ1/N2‐2002, H3‐Cluster IV‐A/N2‐2002, followed by H1‐γ/N1‐classical (Figure 3). This regional analysis also revealed that most of the H1‐δ2 viruses (paired with the 1998‐lineage N2) were detected from Region 1. Importantly, numerically minor genetic clades in the national context were more abundant when considered regionally. For example, the H1‐β viruses were mostly detected in Regions 3 and 4 (4%‐9%), the H1‐α viruses were mostly detected in Regions 1, 2, and 4, the human‐like H3 viruses were mostly detected in Region 2 and 3 (about 9% of IAV in swine from Region 3), and H3‐Cluster IV‐E viruses were only detected in Region 2. Such differences underscore the importance of regional considerations for vaccine development and/or usage.^6^

However, the regional divisions currently implemented for surveillance reporting and described above are unlikely to reflect the realities of swine production systems. Consequently, we used the HA/NA data itself to generate spatial divisions or “zones” (Figure 6B‐E) to provide more insight into swine IAV as it evolves, migrates, and emerges in swine herds. The caveat to this strategy is that the spatial zones indicated by the data here may change with time, and such analyses should be updated with new sequence data on a regular annual basis. The predominant HA/NA pairings in 2016 in zone 1 were H1‐γ/N1‐classical, H1‐δ1/N2‐2002, H3‐human‐like/N2‐2002, and H1‐pdm/N1‐pdm; in zone 2 were H1‐γ/N1‐classical, H1‐δ1/N2‐2002, H1‐δ2/N2‐1998, and H3‐Cluster IV‐A/N2‐2002; in zone 3 were H1‐δ1/N2‐2002, H1‐γ/N1‐classical, H1‐β/N1‐classical, and H3‐Cluster IV‐A/N2‐2002; and in zone 4 were H1‐pdm/N1‐pdm, H3‐Cluster IV‐A/N2‐2002, H1‐δ1/N2‐2002, and H1‐δ2/N2‐1998.

These analyses suggest that licensed multivalent vaccines containing 3‐5 antigens may be regularly updated to target the major viruses circulating in each of these zones. But, additional autogenous or custom vaccines are also likely necessary to effectively target the sustained minor or newly emerging HA/NA pairs. Further complicating vaccine strain selection, HA/NA patterns may also be dependent on specific herd virologic status and pig flow, information which is not shared with the anonymous USDA surveillance system. Vaccination against IAV in swine with killed whole virus vaccines has been employed in the United States for the past 30 years. The general approach for biological companies has been to include strains selected and combine in multivalent form to represent circulating diversity. Unfortunately, due to a paucity of data and a relatively inflexible licensing system, these commercial products were likely to be frequently mismatched between the formulation and the diversity observed in the field.^5^, ^6^, ^47^ Current commercial vaccines typically incorporated predominant genetic clades of H1N1, H1N2, and H3N2 swine viruses circulating in the USA. One commercial vaccine includes an H1‐γ (1A.3.3.3), H1‐δ1 (1B.2.2), H3‐Cluster IV‐A, and H3‐Cluster IV‐B, while another includes an H1‐β (1A.2), H1‐γ (1A.3.3.3), H1‐δ2 (1B.2.1), H3‐Cluster I, and H3‐Cluster IV.^5^, ^48^ A live attenuated influenza A virus vaccine (LAIV) was recently approved for use in the USA, but is not yet in wide‐spread use. As LAIV tend to have broader cross‐protection, it is not clear how many of the current HA/NA clade pairings will be effectively protected against by the viruses in the LAIV or the frequency that the LAIV will need to be updated with the contemporarily relevant dominant viruses. The overall impact of the LAIV on the ecology and evolution of IAV in swine remains to be seen.

The efficacy of vaccination strategies, in addition to homology between the vaccine HA and challenge HA, is also affected by immunogenicity, inoculation route, and other viral genes such as the interplay between the HA and NA. NA antibodies can reduce the severity of infection, hinder the establishment of infections, reduce viral replication, and protect against secondary pathogens.^49^, ^50^, ^51^, ^52^ These studies suggest updating both HA and NA components in IAV vaccines to improve their efficacy. It is reasonable to suggest that a killed vaccine, or autogenous vaccines that are frequently used in the field, may have HA/NA mismatches that will result in vaccine ineffectiveness, complete failure, or vaccine‐associated enhanced respiratory disease.^5^, ^6^, ^53^ Our results showed that across the seven‐year period, the five predominant HA/NA combinations in the United States were H1‐γ/N1‐classical, H1‐δ1/N2‐2002, H3‐Cluster IV‐A/N2‐2002, H1‐δ2/N2‐1998, and H1‐pdm/N1‐pdm, consistent with previous findings.^22^, ^23^ However, changes were observed in the HA/NA diversity across the last 7 years, and within our defined zones. For example, H1‐α/N2‐2002 in Zones 1 and 2, H1‐δ2/N2‐1998 in Zone 2, and H3‐human‐like/N2‐2002 in Zone 1 were more frequently detected in recent years, but are not consistently represented in commercial vaccines. Thus, vaccine strain selection must consider the predominant HA/NA pairings within a zone or production system flow of pigs and be flexible to change. Although we did not have information to connect farms by owner or movement of pigs, spatial clustering of dominant HA/NA pairings at regular timely intervals would help inform strain selection for vaccines and, thus, potentially reduce clinical burden and limit virus spread via increased efficacy of vaccine programs. Antigenic differences among HA and NA clades were not considered in these genetic analyses, but these results also point to dominant HA/NA pairs to target for further antigenic characterization.

The diversity of swine IAV, reflecting spillover of novel viruses from non‐swine hosts and subsequent antigenic drift and shift, represents a considerable problem for swine agriculture and public health. Swine producers and veterinarians voluntarily participated and contributed to the national USDA surveillance system, providing a baseline of information that allows for comparative studies on the genetic diversity of IAV circulating in the swine population between states and over time. This voluntary program and participation is unprecedented globally. These data facilitated the detection of new genetic clades of IAV in swine not effectively controlled by current vaccines^18^, ^22^ and has the potential to inform vaccine development to better control transmission within swine hosts and between swine and humans based on the trends observed here.

## CONFLICT OF INTEREST

The authors declare no conflict of interest.
