# Supplementary material for: Regional patterns of genetic diversity in swine influenza A viruses in the United States from 2010 to 2016
Source: Influenza Other Respir Viruses. 2019 Feb 13;13(3):262–73. doi: 10.1111/irv.12559 (PMC6468071; doi:10.1111/irv.12559)
Supplement: Supplementary file 1 [file IRV-13-262-s001.docx]

**

**Figure S1.** Temporal and regional patterns in subtypes of swine influenza A in the United States. The number of swine isolates within the USDA Swine Influenza A Surveillance is presented by subtype and USDA-APHIS veterinary service district (Region 1 through 4: Region 5 is omitted due to insufficient data) from 2010 to 2016.

**Figure S2.** Representation of hemagglutinin (HA) and neuraminidase (NA) genetic clade pairings found within the USDA Swine Influenza A Surveillance System over all years and all states. The data is presented as percentages, that were calculated based upon phylogenetic analysis and classification of all HA/NA data to HA and NA genetic clade: these data included all available USDA surveillance sequences from 2010 to 2016 from the United States of America.

**Figure S3.** Additive seasonal time series decomposition of all swine IAV isolates in the USDA Swine Influenza A Surveillance system from 2010 to 2016. Observed: data used in the time series analysis was the monthly abundance of all subtyped and sequenced isolates (H1N1, H1N2, H3N2, H3N1); Trend: fitted long-term trend; Seasonal: seasonal component of the time series showing patterns that repeat with fixed period of time; Random: residuals of the time series after allocation into the seasonal and trends time series. The three components (Trend + Seasonal + Random) sum to the time series observed data. The panel scales are not identical.
